# Testis- specific Y-encoded- like protein 1 and cholesterol metabolism: Regulation of *CYP1B1* expression through Wnt signaling

**DOI:** 10.3389/fphar.2022.1047318

**Published:** 2022-11-28

**Authors:** Xiujuan Zhu, Huanyao Gao, Sisi Qin, Duan Liu, Junmei Cairns, Yayun Gu, Jia Yu, Richard M. Weinshilboum, Liewei Wang

**Affiliations:** Department of Molecular Pharmacology and Experimental Therapeutics, Mayo Clinic, Rochester, MN, United States

**Keywords:** TSPYL1, CYP1B1, Wnt, β-catenin, cholesterol, cell signaling, cytochrome P450, obesity

## Abstract

The cytochromes P450 (CYPs) represent a large gene superfamily that plays an important role in the metabolism of both exogenous and endogenous compounds. We have reported that the testis-specific Y-encoded-like proteins (TSPYLs) are novel *CYP* gene transcriptional regulators. However, little is known of mechanism(s) by which TSPYLs regulate *CYP* expression or the functional consequences of that regulation. The *TSPYL* gene family includes six members, *TSPYL1* to *TSPYL6*. However, *TSPYL3* is a pseudogene, TSPYL5 is only known to regulates the expression of *CYP19A1*, and TSPYL6 is expressed exclusively in the testis. Therefore, TSPYL 1, 2 and 4 were included in the present study. To better understand how TSPYL1, 2, and 4 might influence CYP expression, we performed a series of pull-downs and mass spectrometric analyses. Panther pathway analysis of the 2272 pulled down proteins for all 3 TSPYL isoforms showed that the top five pathways were the Wnt signaling pathway, the Integrin signaling pathway, the Gonadotropin releasing hormone receptor pathway, the Angiogenesis pathway and Inflammation mediated by chemokines and cytokines. Specifically, we observed that 177 Wnt signaling pathway proteins were pulled down with the TSPYLs. Subsequent luciferase assays showed that *TSPYL1* knockdown had a greater effect on the activation of Wnt signaling than did *TSPYL2* or *TSPYL4* knockdown. Therefore, in subsequent experiments, we focused our attention on TSPYL1. HepaRG cell qRT-PCR showed that TSPYL1 regulated the expression of CYPs involved in cholesterol-metabolism such as CYP1B1 and CYP7A1. Furthermore, TSPYL1 and β-catenin regulated *CYP1B1* expression in opposite directions and TSPYL1 appeared to regulate *CYP1B1* expression by blocking β-catenin binding to the TCF7L2 transcription factor on the *CYP1B1* promoter. In β-catenin and TSPYL1 double knockdown cells, *CYP1B1* expression and the generation of CYP1B1 downstream metabolites such as 20-HETE could be restored. Finally, we observed that TSPYL1 expression was associated with plasma cholesterol levels and BMI during previous clinical studies of obesity. In conclusion, this series of experiments has revealed a novel mechanism for regulation of the expression of cholesterol-metabolizing CYPs, particularly CYP1B1, by TSPYL1 *via* Wnt/β-catenin signaling, raising the possibility that TSPYL1 might represent a molecular target for influencing cholesterol homeostasis.

## Introduction

The cytochrome P450 (CYP) superfamily consists of a group of enzymes that catalyze the metabolism of numerous endogenous and exogenous compounds including steroids, drugs, carcinogens and natural products (Nebert and Dalton, 2006; Nebert et al., 2013; Nelson et al., 2013). CYPs are expressed in a tissue-specific manner and the expression of many CYPs can be induced by xenobiotics (Esteves et al., 2021). We recently identified members of the testis-specific Y-encoded-like protein (TSPYL) gene family as novel transcription regulators that can influence CYP expression (Liu et al., 2013; Qin et al., 2018; Qin et al., 2020). The *TSPYL* gene family includes six members, *TSPYL1* to *TSPYL6*. Each *TSPYL* gene includes a highly conserved Nucleosome Assembly Protein (NAP) domain but, otherwise, they display relatively little sequence homology (Vogel et al., 1998). NAPs help to assemble DNA and histones reversibly into chromatin, a process that is important for cell proliferation and the regulation of gene expression (Gill et al., 2022). Most TSPYL family members are expressed in all human tissues based on the Genotype‐Tissue Expression (GTEx) database. However, *TSPYL3* is a pseudogene and TSPYL6 is expressed exclusively in the testis. A previous large GWAS study of plasma estradiol concentrations performed by our group reported that single nucleotide polymorphisms (SNPs) in or near *TSPYL5* were associated with plasma estradiol concentrations by virtue of an effect on the expression of CYP19A1—an enzyme critical for estrogen biosynthesis (Liu et al., 2013). Estradiol is synthesized from cholesterol *in vivo* through a series of reactions. CYP19A1 is the only CYP with expression that is currently known to be influenced by TSPYL5 (Qin et al., 2018). Subsequently, TSPYL1, 2 and 4 have all been reported to be involved in the regulation of CYP17A1 and CYP3A4 expression which contributes to abiraterone response in metastatic castration‐resistant prostate cancer (Qin et al., 2018), and the regulation of CYP2C9 and CYP2C19 which affects the metabolism of selective serotonin reuptake inhibitors (Qin et al., 2020). We have also observed that those TSPYLs can regulate the expression of CYP1B1 and CYP7A1 (Qin et al., 2018). However, the consequence(s) of the regulation of CYP1B1 and CYP7A1 have not been explored, and molecular mechanism(s) by which TSPYLs regulate CYP expression remain unknown.

CYP1B1 metabolizes many important physiological compounds, including estrogens, arachidonic acid, melatonin and retinoids (Li et al., 2017). Hydroxyeicosatetraenoic acids (HETEs), including 20-HETE and 12-HETE, are the major CYP1B1 arachidonic acid metabolites in humans (Choudhary et al., 2004). Clinical studies have reported elevated levels of plasma and urinary 20-HETE in disease states that include obesity and CYP1B1 knock out in mouse models has been reported to protect against obesity induced by a high-fat diet (Li et al., 2014; Larsen et al., 2015) and lack of CYP1B1 is linked to altered lipid metabolism, an association which may help protect against the negative health effects of obesity.

CYP7A1 catalyzes the initial, rate-limiting step in the bile acid biosynthetic pathway. In mammals, excess cholesterol in the liver is removed mainly by conversion to bile acids. Only a small portion of cholesterol is utilized for steroid hormone synthesis in the adrenal glands, ovaries, testes, placenta and brain. Transgenic mice overexpressing CYP7A1 are resistant to high fat diet-induced obesity, fatty liver and diabetes (Li et al., 2010). Based on this series of observations, the present study has focussed on the possible role of TSPYL1 in cholesterol biosynthesis and metabolism because, as explained subsequently, TSPYL1 displayed a greater effort on Wnt signaling than did TSPYL2 or TSPYL4. Specifically, we found that TSPYL1 interacted with proteins in the Wnt/β-catenin signaling pathway. Wnt/β-catenin signaling determines hepatic zonation of CYP expression (Loeppen et al., 2005; Hailfinger et al., 2006; Sekine et al., 2006; Braeuning and Schwarz, 2010) and is involved in the regulation of CYP transcription in response to exposure to xenobiotic agonists for a number of nuclear receptors (Braeuning et al., 2009; Ganzenberg et al., 2013; Vaas et al., 2014). Specifically, β-catenin is a downstream target of Wnt signaling (Kimelman and Xu, 2006). In the nucleus, β-catenin binds to transcriptional activators of the T-cell factor/lymphoid-enhancing factor (TCF/LEF) family to activate the transcription of Wnt signaling target genes.

Wnt/β-catenin signaling plays an important role in obesity (Wang et al., 2013) as does the TCF/LEF family. This family of transcription factors includes TCF7, LEF1, TCF7L1, and TCF7L2. As described subsequently, we found that both TCF7 and TCF7L2 were also pulled down with TSPYLs (see Supplementary Table S1). Genetic polymorphisms in the *TCF7L2* gene have been associated with obesity and increased BMI (Cauchi et al., 2008; Haupt et al., 2010). Although the regulation of CYPs by Wnt/β-catenin signaling and by TSPYL1 were known, potential interaction of β-catenin with TSPYL1 had not previously been described. Therefore, included among the goals of the present study was a determination of whether Wnt/β-catenin signaling had an effect on the regulation of CYP expression by TSPYL1 in human hepatic cell lines. We hypothesized that TSPYL1 might regulate CYP expression through interaction with the Wnt/β-catenin signaling pathway. Our results—as described subsequently--demonstrated that TSPYL1 can compete with TCF7L2 for binding to β-catenin, reducing Wnt/β-catenin activity. Taken together, these results suggest that variation in TSPYL1 expression, Wnt/β-catenin signaling and CYP expression may all contribute to risk for or variation in response to the treatment of obesity.

## Materials and methods

### Cell culture and transfection

Undifferentiated HepaRG human hepatic cells (HPR101) were obtained from Biopredict (Rennes, France) and were cultured and differentiated into fully functional hepatocyte-like cells according to the manufacture’s protocol. HPR101 cells were grown in William’s E media (Gibco, Grand Island, NY) supplemented with ×1 GlutaMAX (Gibco, Grand Island, NY) and 10% HepaRG Growth Medium Supplement (Biopredict, Rennes, France) until confluent. Cells were then switched into HepaRG Differentiation media for 2 weeks. Differentiated HepaRG cells were used directly for transfection.

HepG2 cells (the American Type Culture Collection, ATCC) were grown in Eagle’s Minimum Essential Medium containing 10% fetal bovine serum (FBS) (HyClone). HEK 293T were grown in Dulbecco’s modified Eagle’s medium containing 10% FBS. STF cells (ATCC) were cultured in 80% DMEM F12 Medium, (ATCC 30-2006), add 20% Bovine Calf Serum, Iron Fortified (ATCC 30-2030) and 200ug/ml G-418. All cells were cultured at 37°C with 5% CO_2_ and used within 30 passages. Cells were transfected with plasmids using lipofectamine 2000 (Thermo scientific). Cells were transfected with siRNA using Lipofectamine RNAiMAX (Invitrogen). Specifically, for 6 well plates, each well used 4 µl Lipofectamine RNAiMAX reagent and 4 µl siRNA (10 µM).

### Real-time quantitative reverse transcription polymerase chain reaction

Total RNA was purified using the QIAGEN RNeasy kit (Germantown, MD). Primers for the amplification of TSPYL1, CTNNB1 and CYPs were Prime Time pre-designed qPCR primers (IDT Inc., Coralville, Iowa). All samples were measured in technical triplicates using the SYBR green reagent with an ABI Prism 7000HT sequence detection system (Applied Biosystems). The results were quantified using the Comparative Ct (ΔΔCt) method with the housekeeping gene GAPDH as an internal reference control.

### Western blot analysis

Protein levels were determined using Western blot analysis. The following primary antibodies were used: Flag (1:1,000, Cell Signaling Technology, cat. #F1804), GAPDH (1:1,000, Cell Signaling Technology, cat. #5174S), β-catenin (1:1,000, Cell Signaling Technology, cat. #9582S), TSPYL1 (1:1,000, Bethyl Laboratories, cat. #A304-852A-M), and TCF7L2 (1:1,000, Cell Signaling Technology, cat. #2569S). The following secondary antibodies were used: Peroxidase- conjugated AffiniPure Goat Anti-Mouse IgG, light chain specific (1:2,000 dilution, Jackson immunoRsearch) and Peroxidase- conjugated IgG fraction monoclonal mouse anti-rabbit IgG, light chain specific (1:2,000 dilution, Jackson Immuno Research).

### IP and IB analysis

For Co-IP, 293T cells were grown in 10-cm dishes and were transfected with the appropriate plasmids for 48 h. Cell lysates were incubated with 3 μg of Flag antibody on a rotator overnight at 4°C. The protein–antibody–protein A/G-agarose complexes were prepared by adding 50 μl of protein A/G-agarose beads (Thermo scientific) for 2 h at 4°C. After three washings with NETN lysis buffer, the immunoprecipitated complexes were resuspended in reducing sample buffer and elute at 50°C for 10 min. The supernatants were transferred to a clean tube and boiled for 5 min. Supernatants were subjected to SDS–polyacrylamide gel electrophoresis (PAGE) and IB.

### Mass spectrometry

Mass Spectrometry was done using differentiated HepaRG cells with IRES-TSPYL1, 2 or 4 plasmids transiently transfected using lipofectamine 2000 for 48 h. TSPYL interacting proteins were immunoprecipitated using Flag antibody. Normal mouse IgG was used as a negative control. The immunoprecipitated protein was eluted using 2X Laemmili buffer and resolved using SDS-PAGE electrophoresis. Protein bands were visualized by Coomassie Blue Staining and were dissected into sections equally. The dissected gel slices were sent for Mass spectrometric analysis at the Harvard Taplin Mass Spec facility. Proteins uniquely precipitated using Flag antibody but not IgG, and shared among all three TSPYLs were included for further pathway analysis. Pathway analysis was performed using the Panther pathway database.

### Clinical data

TSPYL1 expression, cholesterol and BMI data from patients were downloaded from the Gene Expression Omnibus (https://www.ncbi.nlm.nih.gov/geo/) by GEO ID GSE48452 and GSE130991. Probe IDs were labeled in the figure legends. For the BMI analysis, samples were divided by TSPYL1 median expression level. The GSE130991 dataset was used for the age-specific LDL-cholesterol level test. Samples were grouped by both the median expression of TSPYL1 and the population median age. Statistical significance was tested by the Mann-Whitney test.

### Luciferase assays

SuperTopFlash HEK293 cell line (STF) reporter cells were split into 24-well plates and were transfected 24 h later with 1ug of TSPYL overexpression plasmid or siRNA and 0.8 ng of the transfection control Renilla luciferase plasmid pTK-RL (Promega) using lipofectamine 2000 (Thermo scientific). 48 h after transfection, the cells were washed with PBS and luciferase activities were measured with a Dual-Luciferase Assay Kit (Promega, cat# E1960) according to the manufacturer’s protocols. Luciferase assays were performed at least in triplicate.

### Plasmids and siRNAs and reagents

SMARTpool siRNA duplexes specific for *CTNNB1* (Catalog ID: M-003482-00-0005), *TSPYL1* (Catalog ID: M-028592-01-0005), *TSPYL2* (Catalog ID: M-013880-01-0005), *TSPYL4* (Catalog ID: M-017980-00-0005), and a Non-Targeting siRNA pool (siCTRL, Catalog ID:D-001206-13-05) were purchased from Dharmacon. Recombinant human Wnt-3a was purchased from R&D Systems. Human TSPYL cDNA constructs and empty vector, Pcmv6-XL4, were purchased from Origene Technologies (Rockville. MD).

### CHIP assays

HepaRG cells were used to perform ChIP assays to determine possible TSPYL1 binding to the promoter regions of *CYP1B1* or *CYP7A1*. Primer sets (see Supplementary Table S2) which covered TCF7L2 binding sites of *CYP1B1* or TCF7 binding sites of *CYP7A1*, were designed to “screen” potential TSPYL1-DNA binding sites. ChIP assay was performed using the SimpleChIP Enzymatic Chromatin IP kit (Cat. #91820, cell signaling technology) followed by quantitative PCR using the TB Green Premix Ex Taq™ PCR master mix reagent (Cat. #RR420A, TaKaRa).

### Determination of 20-hydroxyeicosatetraenoic acid levels

The concentration of 20-HETE was determined by an ELISA kit (20H39-K01, Eagle Biosciences) according to the manufacturer’s instructions. For measurement of 20-HETE in cultured HepaRG cells, cells were trypsinized in 0.05% trypsin after washing with PBS. Half of the cells were used for PCR. The remainder of the cells were homogenized and sonicated with RIPA buffer containing triphenylphosphine. 50 µl was used for the 20-HETE assay on the day of extraction. The remaining homogenate was frozen at −80 C for protein assay.

### Statistics

A two-tailed Student-*t* test was used for statistical analysis for changes across conditions, if not specified. A *p value* < 0.05 was considered to be statistically significant. Statistical significance is indicated by asterisks. *p < 0.05; **p < 0.01; ***p < 0.001, ****p < 0.0001.

## Results

As stated above, we previously identified TSPYLs as novel CYP gene transcriptional regulators (Qin et al., 2018; Qin et al., 2020). However, relatively little is known with regard to mechanisms by which TSPYLs might regulate CYP expression or how their effect on CYP transcriptional regulation might influence human biology and/or pathophysiology. The studies described subsequently revealed a novel mechanism for regulation of the expression of cholesterol-metabolizing CYPs, particularly CYP1B1, by TSPYL1, in a process mediated by Wnt/β-catenin signaling. These observations also raised the possibility that TSPYL1 might be a novel target for drugs designed to influence cholesterol homeostasis.

### TSPYLs interact with Wnt signaling pathway proteins

**FIGURE 1:**
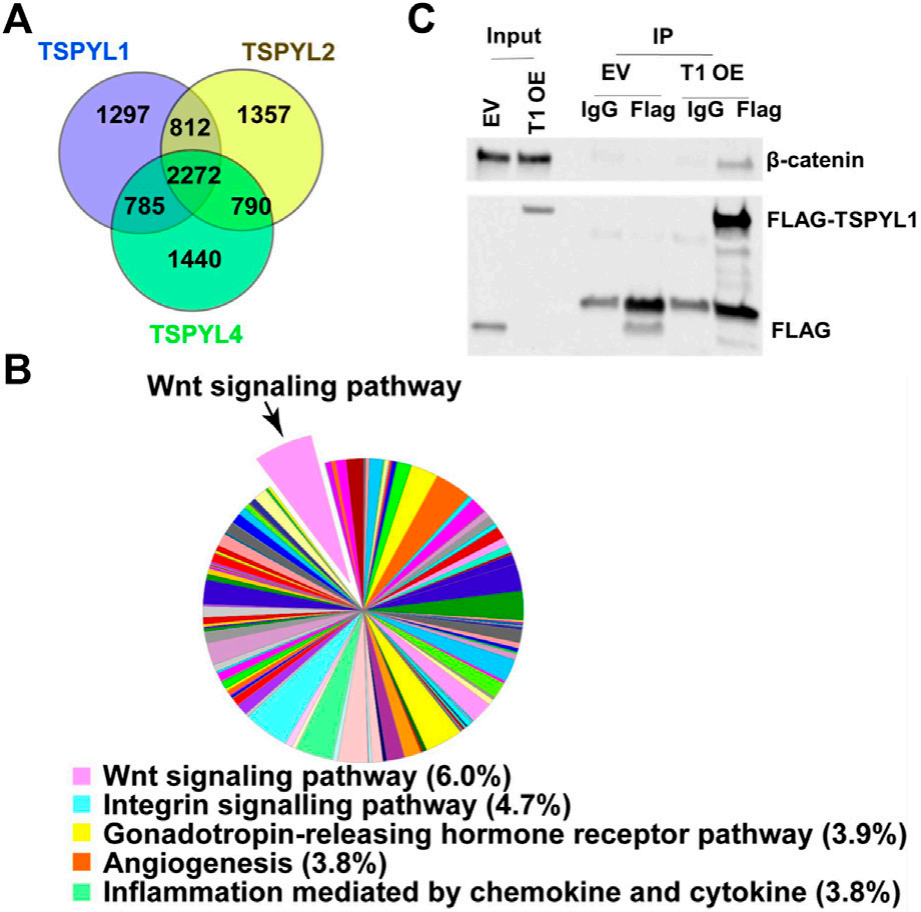
TSPYL1,2 and 4 interact with Wnt signaling pathway proteins. **(A)** Venn diagram depicting the number of proteins pulled down by TSPYL1 (blue circle), TSPYL2 (yellow circle), or TSPYL4 (green circle) respectively and identified by mass spectrometry in HepaRG cells. **(B)** Signaling pathway analysis of the 2272 proteins pulled down by all 3 TSPYL isoforms. A pie chart showing the quantity of proteins matched with Panther pathways. The top five pathways are shown in the legend with percentages of input genes noted after the pathway name. The Wnt signaling pathway is shown as a pop-out slice. **(C)** Co-IP of FLAG-TSPYL1 with endogenous β-catenin in 293T cells. FLAG-TSPYL1 was immunoprecipitated, and the quantity of β-catenin bound to TSPYL1 was determined using immunoblot with an anti-β-catenin antibody. The quantities of TSPYL1 and β-catenin immunoprecipitated were compared with IgG. (EV- empty vector; T1 OE- TSPYL1 over expression).

To study mechanisms and potential signaling pathways that interact with TSPYLs, we performed flag-affinity purification of 3XFLAG-TSPYL1, 2 and 4 from HepaRG cells after IRES plasmid transient transfection to isolate TSPYL1,2 and 4-associated proteins. A total of 2272 proteins were pulled down for these three TSPYL isoforms as shown by the Venn diagram in Figure 1A. The pie charts and Venn diagram of panther pathway analysis for each individual pull down by TSPYL1, 2 and 4 are shown in Supplementary Figure S1. Panther pathway analysis was performed for these 2272 proteins. Included among the top signaling pathways associated with TSPYLs on the basis of mass spectrometric analysis were Wnt signaling pathway proteins (Figures 1A,B). The other top 4 pathways were the Integrin signaling pathway, the Gonadotropin- releasing hormone receptor pathway, the Angiogenesis pathway, and Inflammation mediated by chemokines and cytokines. All of the protein pull-down information is available in a Supplementary Excel File.

β-Catenin is a key component of Wnt canonical signaling (Mosimann et al., 2009). It is known that β-catenin binds to TCF/LEF family members to activate the transcription of Wnt signaling target genes (Mosimann et al., 2009). Therefore, we confirmed the mass spectrometric results by performing co-immunoprecipitation using 293T cells and found that endogenous β-catenin (Figure 1C) could be co-precipitated with Flag-tagged-TSPYL1.

### TSPYL1 knockdown increased Wnt signaling activity

**FIGURE 2:**
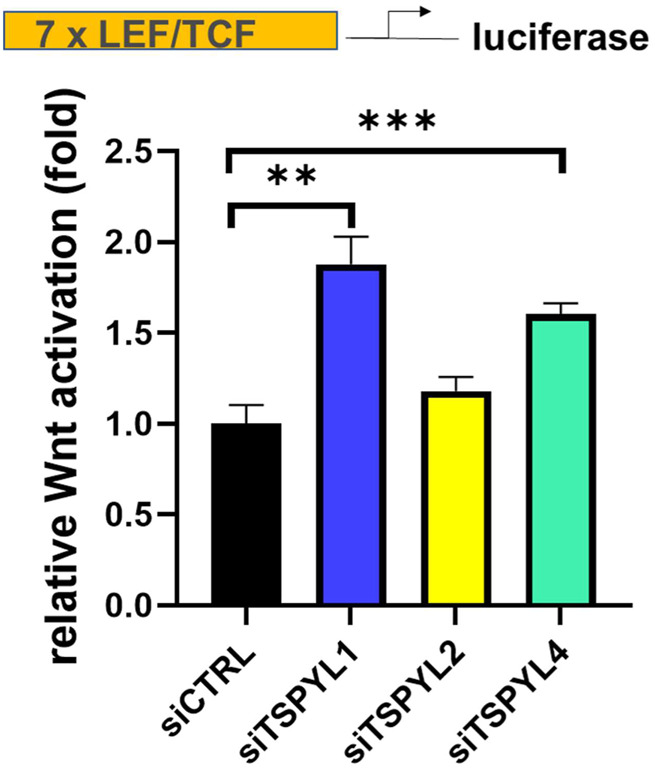
TSPYL1,2 and 4 regulate Wnt signaling activity. Knockdown of TSPYL1, or TSPYL4 resulted in an increase in Wnt signaling activity. Luciferase assays using a SuperTopFlash HEK293 cell line (STF reporter cells) which contains a luciferase reporter under the control of 7 LEF/TCF binding sites, utilizes the SuperTopFlash system, with or without TSPYL1, 2, or 4 knock down. Error bars indicate standard deviations among 3 technical replicates per experiment. Data are represented as mean ± SD. (*p < 0.05, **p < 0.01, ***p < 0.001).

Because of the nuclear localization and the association among TSPYLs, β-catenin and TCF/LEF, we next tested the possibility of a functional link between TSPYLs and Wnt signaling. We used SuperTopFlash (STF) HEK293 reporter cells to determine whether the knockdown of TSPYLs could affect Wnt signaling. TSPYL1 knockdown increased luciferase reporter transcription (Figure 2). TSPYL2 and TSPYL4 knockdown also increased Wnt activity 1.25 and 1.55 fold, respectively, but less than that observed for TSPYL1 (1.73 fold) (Figure 2). These observations raised the possibility that these TSPYLs might be negative regulators of Wnt/β-catenin signaling.

### TSPYL1 knockdown regulates cholesterol metabolizing CYPs

**FIGURE 3:**
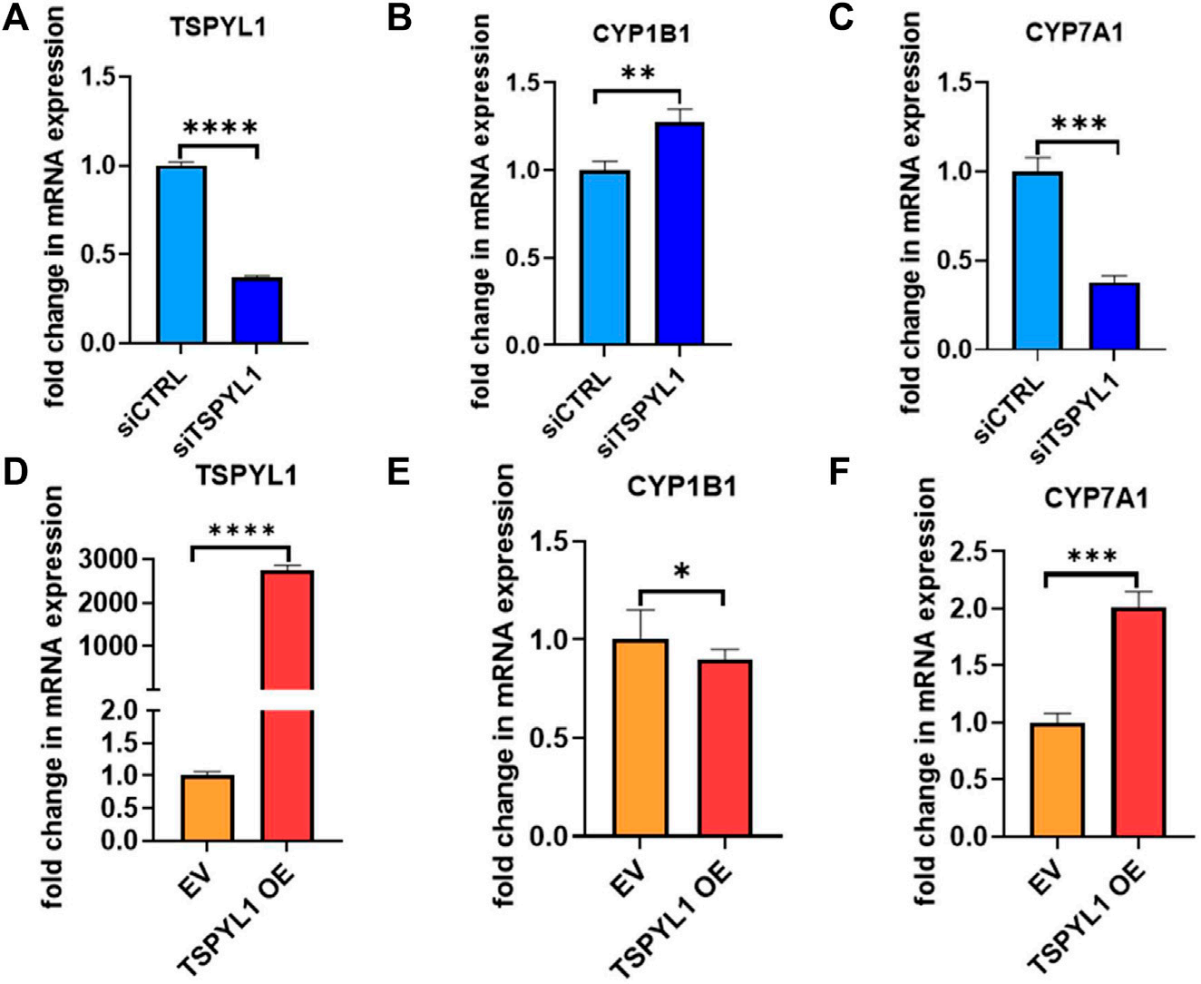
TSPYL1 regulates *CYP1B1* and *CYP7A1* expression in HepaRG cells. **(A)** HepaRG cells with or without TSPYL1 depletion by siRNA were transfected as indicated. *CYP1B1* **(B)** and *CYP7A1* **(C)** expression were tested by qRT-PCR. **(D)** HepaRG cells with or without TSPYL1 overexpression were transfected as indicated. *CYP1B1* **(E)** and *CYP7A1* **(F)** expression were tested by qRT-PCR. Gene-specific data were normalized to GAPDH expression. Error bars indicated standard deviations among 3 technical replicates per experiment. Data are represented as mean ± SD. (*p < 0.05, **p < 0.01, ***p < 0.001, ****p < 0.0001).

**FIGURE 4:**
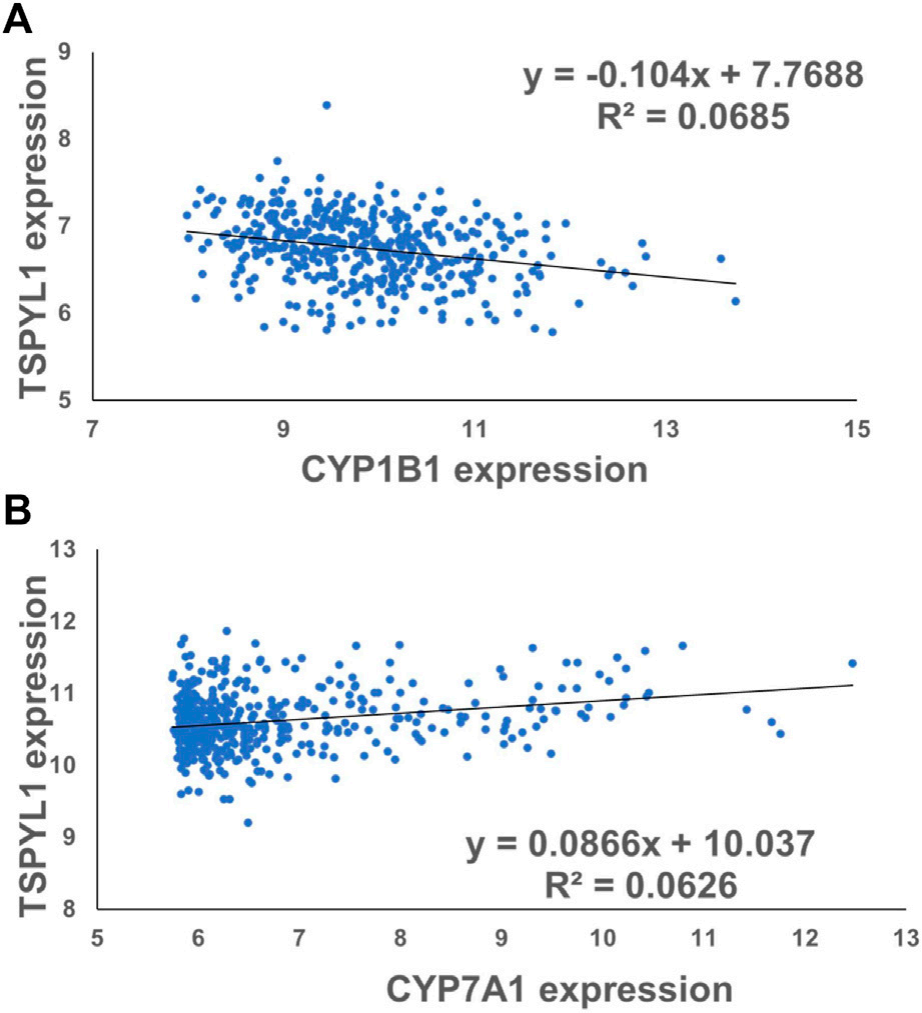
TSPYL1 expression associates with CYP1B1 and CYP7A1 expression levels in human liver tissue. **(A)** The expression correlation coefficient for CYP1B1 and TSPYL1 was −0.26. (r = −0.26, ****,*p* = 1.35e-08). **(B)** The expression correlation coefficient for CYP7A1 and TSPYL1 was 0.25. (r = 0.25, ****, *p* = 5.54e-08). These data were obtained from a previous publication Innocenti et al. (2011).

CYPs play an important role in the maintenance of cholesterol homeostasis (Pikuleva, 2006). For example, CYP7A1 is the first and the rate-limiting enzyme in the classic bile acid synthesis pathway in which cholesterol is metabolized to form bile acids, while CYP11A1 catalyzes a step in a pathway by which cholesterol serves as a substrate for the synthesis of steroid hormones (Pikuleva, 2006). CYP1B1 also has a significant impact on cholesterol metabolism. Consistent with our previous observations (Qin et al., 2018), we found that CYP1B1 was upregulated by TSPYL1 knockdown (Figures 3A,B) and that it was downregulated by TSPYL1 overexpression in HepaRG cells (Figures 3D,E). CYP7A1 was significantly downregulated by TSPYL1 knockdown (Figure 3C) and was upregulated by TSPYL1 overexpression in those cells (Figure 3F). PCR results had previously shown that CYP11A1 was not significantly regulated by TSPYL1 knockdown (Qin et al., 2018), so we did not perform follow up studies on CYP11A1. We next analyzed the correlation of TSPYL1 and CYP1B1 expression, using expression quantitative trait loci (eQTL) data from human hepatic tissue (Innocenti et al., 2011). The expression of TSPYL1 and CYP1B1 were negatively correlated, with a correlation coefficient of -0.26 (Figure 4A, *p* = 1.35e-08). When we analyzed the correlation of TSPYL1 with CYP7A1 expression, we observed that TSPYL1 and CYP7A1 expression were positively correlated, with a correlation coefficient of 0.25 (Figure 4A, *p* = 5.54e-08).

### TSPYL1 and TCF7L2 bind to the promoter region of CYP1B1 in HepaRG cells

**FIGURE 5:**
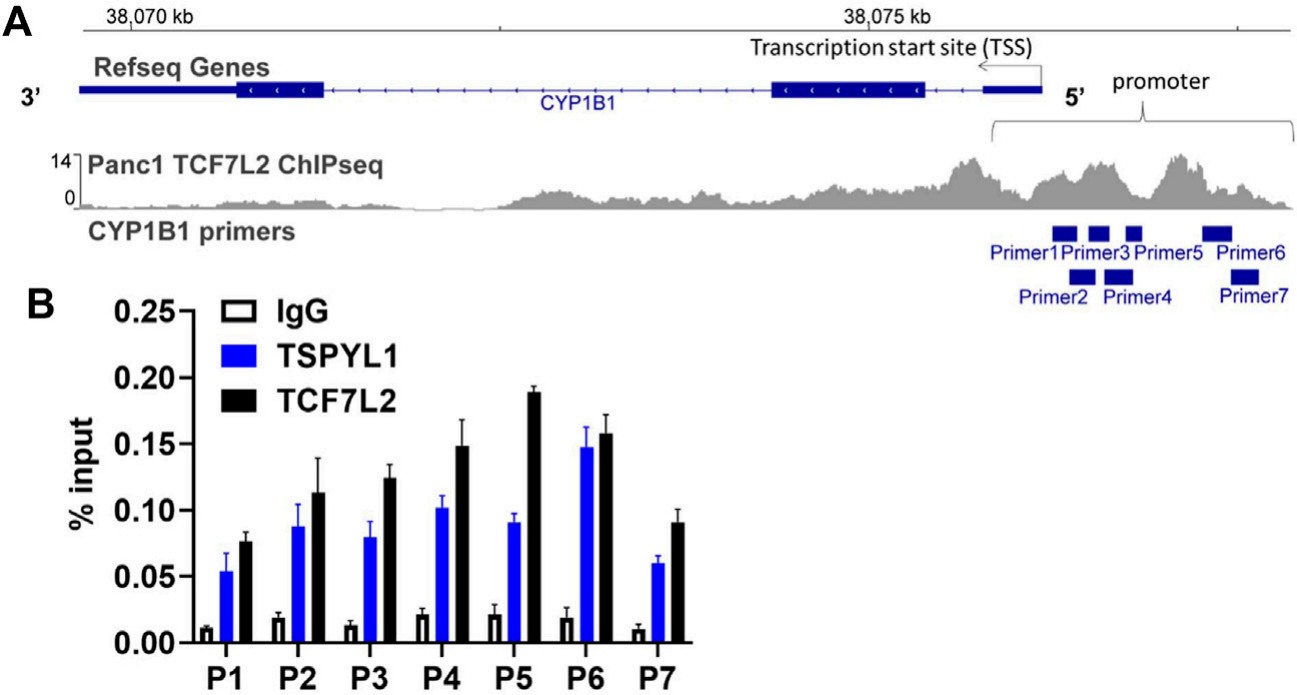
TSPYL1 and TCF7L2 bind to the promoter region of *CYP1B1* in HepaRG cells. **(A)** Seven pairs of primers covering the *CYP1B1* promoter region were used to perform CHIP assays. **(B)** CHIP assays were performed to test TSPYL1 and TCF7L2 binding to the *CYP1B1* promoter region using HepaRG cells. The binding of TSPYL1 and TCF7L2 to *CYP1B1* promoter regions was detected by qRT-PCR and has been expressed as percent of input.

**FIGURE 6:**
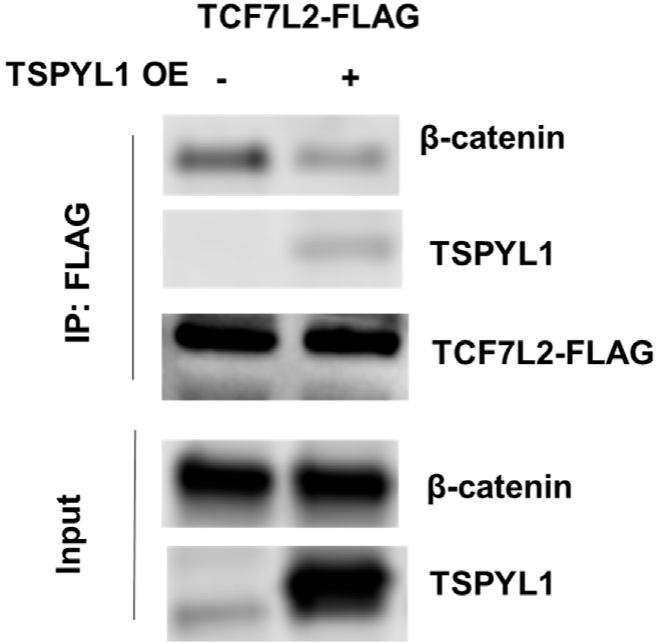
TSPYL1 blocks β-catenin binding with TCF7L2. Interaction of endogenous β-catenin with overexpressed TSPYL1 and TCF7L2-FLAG is shown. IP was conducted with whole-cell lysates of 293T cells transfected with TCF7L2-FLAG and an empty vector or TSPYL1 overexpression plasmid. TSPYL1 overexpression reduced β-catenin binding with TCF7L2.

According to the TCF7L2 ChIP-seq data in the ENCODE database, TCF7L2 binds to the *CYP1B1* promoter in Panc1 cells (Figure 5A). We hypothesized that TSPYL1, by interacting with β-catenin and TCF7L2, might bind to the same *CYP1B1* promoter region as does TCF7L2 in HepaRG cells. Therefore, we performed CHIP assays with antibodies to both TSPYL1 and TCF7L2 using HepaRG cells because of the high protein expression level of TSPYL1 in HepaRG cells. Specifically, a series of PCR primers was designed to amplify *TCF7L2* binding sites in the *CYP1B1* promoter. Our CHIP results showed that TSPYL1 bound to the same *CYP1B1* promoter region as did TCF7L2 (Figure 5B). We next performed co-immunoprecipitation to study possible interactions among TCF7L2-FLAG, TSPYL1 and endogenous β-catenin. The results showed that TSPYL1 overexpression dramatically reduced β-catenin binding to *TCF7L2* (Figure 6). These results suggested that the expression of TSPYL1 might influence the interaction between TCF7L2 and β-catenin, specifically that TSPYL1 might interfere with β-catenin binding to TCF7L2.

A putative bile acid responsive element, BAREII (DR1), is present in the human *CYP7A1* gene promoter. TCF7 binds to BAREII to regulate CYP7A1 gene transcription in HepG2 cells based on ENCODE data (see Supplementary Figure S2A). Therefore, we performed a CHIP assay to determine whether TSPYL1 might bind to the BAREII element in the *CYP7A1* promoter. Four pairs of primers for PCR of the *CYP7A1* promoter region were used. We failed to detect significant TSPYL1 recruitment to the *CYP7A1* promoter region in HepaRG cells (see Supplementary Figure S2B).

### CYP expression regulation by Wnt signaling in HepG2 cells

The effect of the WNT/β-catenin pathway on the regulation of the expression of major human P450 enzymes in HepaRG cells has been studied extensively (Thomas et al., 2015), but CYP1B1 had not been studied in that context. To obtain additional information on the effect of the WNT/β-catenin pathway in hepatic cells, we studied an additional cell line, HepG2. To identify CYP genes that were transcriptionally regulated by Wnt, we determined the effect of Wnt pathway activation or inhibition on the expression of a series of CYPs in HepG2 cells. As a first step, we compared the effect of β-catenin siRNA and control siRNA knockdown on CYP expression. siRNA-mediated knockdown of β-catenin resulted in an approximate 75% decrease in the expression of β-catenin (*CTNNB1*) mRNA and its downstream target genes (see Supplementary Figure S3C). We also tested the effect of stimulation with the canonical Wnt ligand, Wnt-3a. Wnt-3a treatment significantly induced the expression of Wnt signaling downstream genes compared with BSA treatment as a control (see Supplementary Figure S3D). Changes in CYP mRNA expression in HepG2 cells were also determined. Specifically, PCR analysis revealed that CYP1B1, CYP2A6 and CYP2B6 were transcriptionally downregulated more than 2-fold by β-catenin knockdown in HepG2 cells (see Supplementary Figure S3A), whereas expression of CYP2C9, CYP2C18, CYP3A7, CYP7A1, CYP7B1, and CYP27A1 were upregulated by β-catenin knock down in HepG2 cells (see Supplementary Figure S3A). By contrast, CYP1B1, CYP2A6 and CYP2B6 expression was significantly upregulated after Wnt-3a stimulation, while the expression of CYP2C9, CYP2C19 and CYP3A7 was downregulated more than 2-fold by Wnt activation (see Supplementary Figure S3B). These results support the conclusion that the expression of CYP1B1, together with that of other CYPs, is regulated by Wnt/β-catenin signaling in HepG2 cells.

### *CYP1B1* expression was restored in β-catenin and TSPYL1 double knockdown HepaRG cells

**FIGURE 7:**
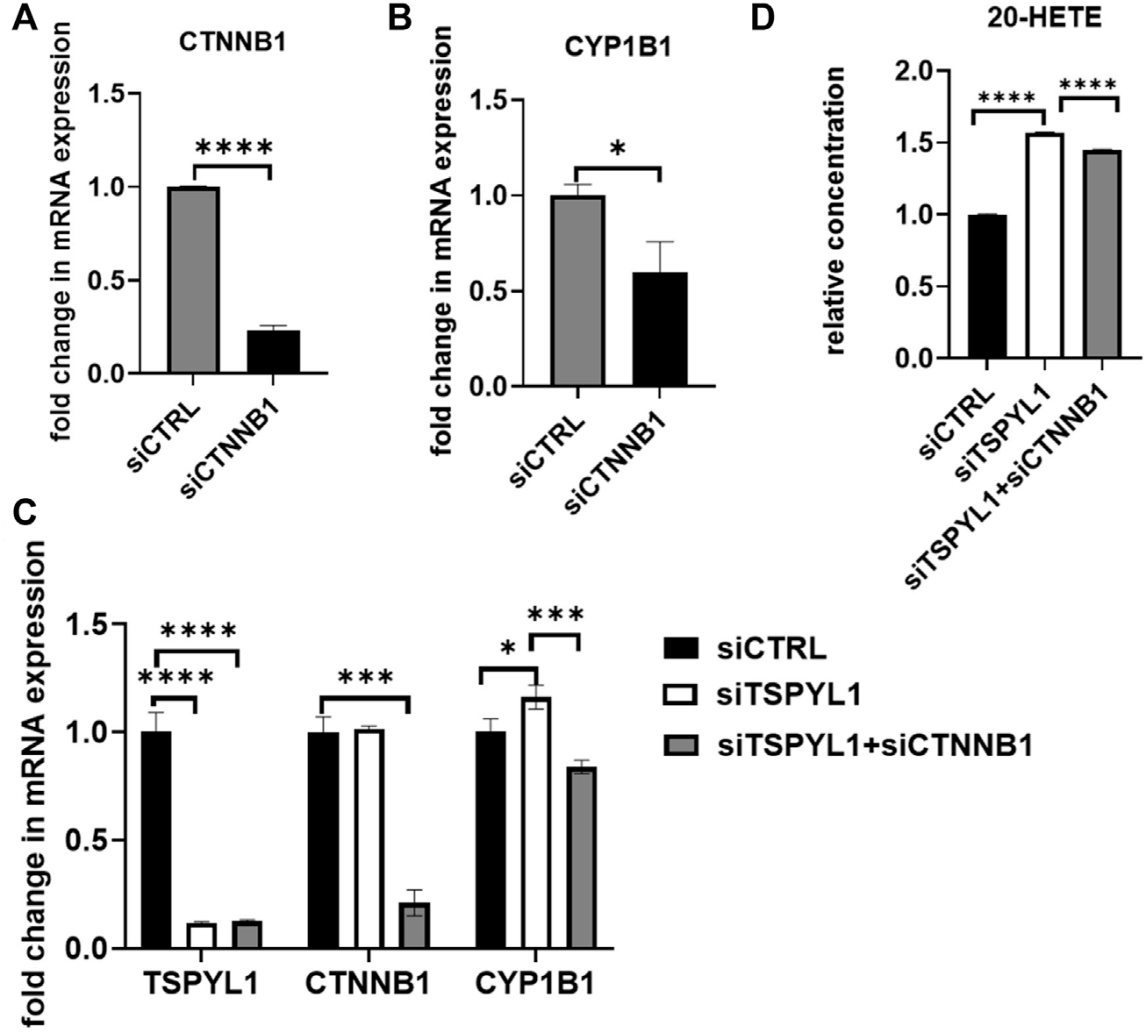
β-Catenin knockdown rescues TSPYL1 knockdown-related increase in CYP1B1. **(A)** HepaRG cells with or without β-catenin (CTNNB1) depletion by siRNA were transfected as indicated. **(B)** *CYP1B1* expression was tested by qRT-PCR. **(C)** HepaRG cells with TSPYL1 or CTNNB1 depletion by siRNA were transfected as indicated. *CYP1B1* expression was tested by qRT-PCR. Gene-specific data were normalized to GAPDH expression. **(D)** 20-HETE was measured by ELISA. Error bars indicate standard deviations among 3 technical replicates per experiment. Data are represented as mean ± SD. (*p < 0.05, **p < 0.01, ***p < 0.001, ****p < 0.0001).

We had demonstrated that TSPYL1 negatively regulated *CYP1B1* expression by blocking β-catenin binding to TCF7L2 on the *CYP1B1* promoter. After β-catenin knockdown, *CYP1B1* expression was down regulated in HepaRG cells (Figures 7A,B). In summary, TSPYL1 and β-catenin knockdown regulated *CYP1B1* expression in opposite directions.

Therefore, we anticipated that by inhibiting the activity of the Wnt/β-catenin pathway, we could “rescue” TSPYL1 knockdown-dependent *CYP1B1* upregulation. To test that hypothesis, we conducted a double knockdown experiment. Our PCR results supported the hypothesis that *CYP1B1* expression could be rescued by β-catenin and TSPYL1 double knockdown (Figure 7C). 20-HETE, a downstream metabolite of CYP1B1, was also restored by TSPYL1 and CTNNB1 double knockdown (Figure 7D). These results supported the conclusion that TSPYL1 regulates CYP1B1 expression through Wnt/β-catenin signaling.

### TSPYL1 has been associated with BMI and cholesterol levels in patients with obesity

**FIGURE 8:**
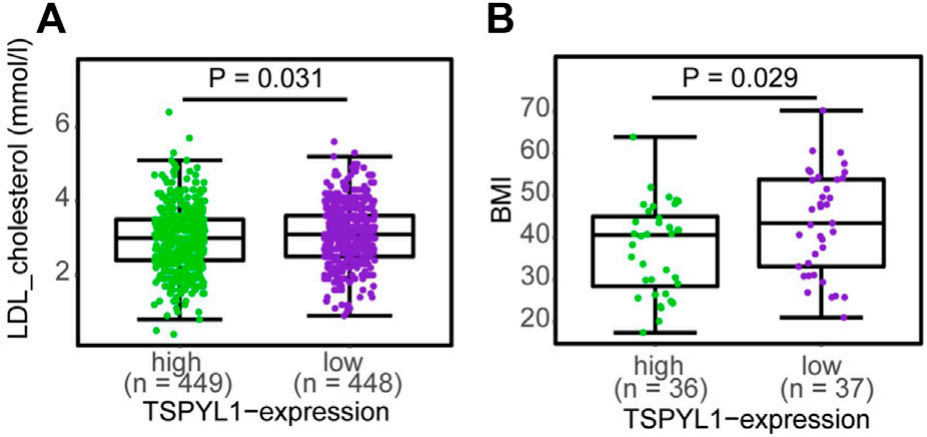
TSPYL1 expression and cholesterol levels and BMI in clinical trials. **(A)** LDL-cholesterol levels subgrouped by median TSPYL1 expression, using hepatic expression array data for the GSE130991 dataset. **(B)** BMI measurements grouped by median TSPYL1 expression using data from the GSE48452 dataset. Statistical significance was tested by use of the Mann-Whitney test with *p*-values indicated above the figures.

Transgenic mice overexpressing CYP7A1 in the liver have been reported to be resistant to high-fat diet induced obesity, fatty liver disease and diabetes (Li et al., 2010), while decreased CYP1B1 expression has been correlated with altered lipid metabolism, especially lysophosphatidylcholines, contributing to protection against the development of obesity (Li et al., 2014). Since TSPYL1 knockdown in hepatic cells resulted in increased CYP1B1 and decreased CYP7A1 expression, we asked whether TSPYL1 expression might be associated with obesity and/or plasma cholesterol concentrations in humans. As an initial step toward systematically testing the hypothesis that hepatic TSPYL1 expression might be associated with obesity, we examined publically available data from two clinical studies addressing obesity in which hepatic mRNA expression had been determined. The first trial entitled “A Biological Atlas of Severe Obesity (Biological Tissue Collection (ABOS)” (ClinicalTrials.gov Identifier: NCT01129297) involved 897 obese patients. Figure 8A shows the results of transcriptomic profiling of hepatic tissue from those 897 obese patients (Mean BMI 46.7). Among those patients, 173 received statin treatment, and differential expression analysis was performed between samples from patients who were treated with or without statins to test possible statin effects on gene expression. TSPYL1 expression levels for those patients were negatively associated with plasma LDL-cholesterol levels. A separate cohort from the “Human Liver Biopsy of Different Phases of Control to NASH” (GSE48452) study involved 73 severely obese patients with various degrees of non-alcoholic fatty liver disease (NAFLD). Those patients also had transcriptomic profiling of hepatic tissue, and hepatic TSPYL1 expression in this group was negatively associated with BMI (Figure 8B). Both of these datasets were analyzed using non-parametric two-group testing (Mann-Whitney test). These two examples are, of course, merely suggestive, and studies designed to specifically test hypotheses arising from the present series of studies will have to be performed in the future.

## Discussion

The results of the experiments described above indicate that TSPYL1 represents one factor regulating the expression of CYPs including, especially, CYP1B1, with a significant effect on cholesterol concentrations. This effect of TSPYL1 appears to be mediated, at least in part, through Wnt/β-catenin signalling. These observations suggest, among other implications, that TSPYL1 might represent a target for future attempts to influence or modify cholesterol biosynthesis.

**FIGURE 9:**
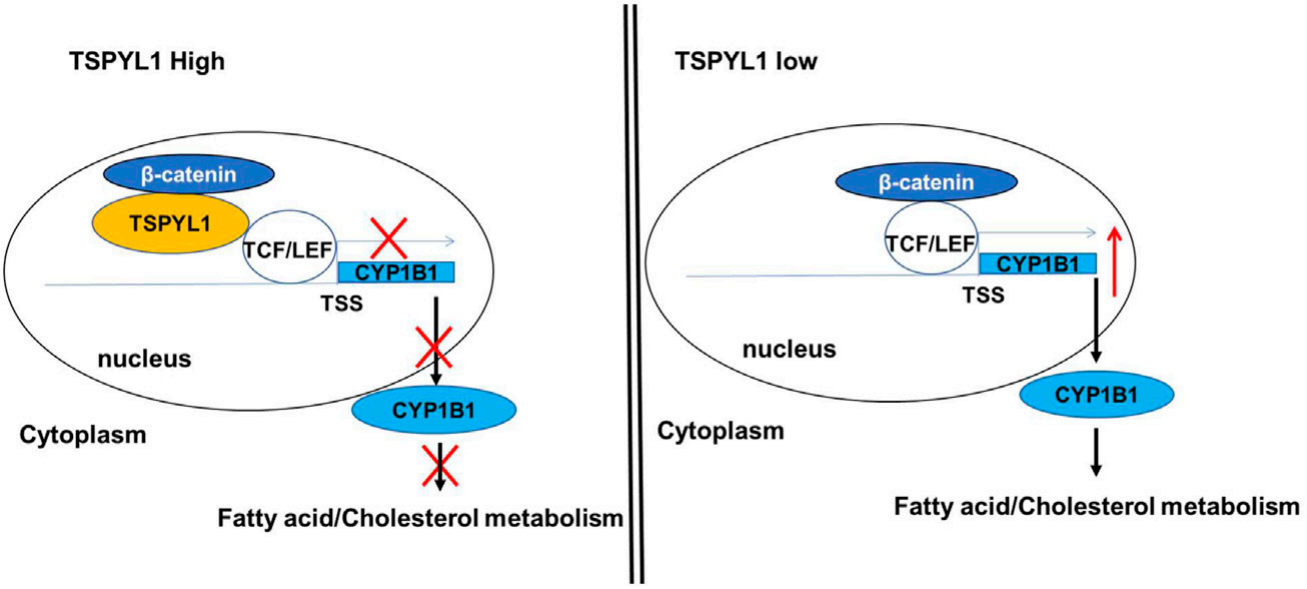
Proposed schematic model for the role of TSPYL1 in *CYP1B1* transcription regulation. β-Catenin is a transcriptional activator for *CYP1B1*. TSPYL1 inhibits β-catenin binding with TCF/LEF on the *CYP1B1* promoter. When the TSPYL1 level decreases, β-catenin binds to TCF/LEF in the nucleus. This activates *CYP1B1* transcription and interferes with fatty acid and cholesterol metabolism.

As shown schematically in Figure 8A, decreased TSPYL1 expression is associated with elevated LDL-cholesterol plasma levels in patients. This relationship can be explained, at least in part, by the model shown in Figure 9. When TSPYL1 levels decrease, β-catenin binds to TCF/LEF in the nucleus. That binding activates *CYP1B1* transcription which may then alter fatty acid and cholesterol metabolism.

Specifically, our observations raise the possibility that CYP1B1 could represent a therapeutic target for the treatment of selected metabolic diseases. CYP1B1 knockdown altered the expression of 560 hepatic genes, including the PPARs, a group of nuclear receptors that play a key role in lipid and glucose homeostasis (Larsen et al., 2015). Metabolic pathways regulated by CYP1B1 include steroid hormone metabolism, fatty acid metabolism, vitamin metabolism and melatonin metabolism (Li et al., 2017). *CYP1B1* is an important gene associated with obesity, based on a review of 49 obesity-related genome-wide sequencing studies covering 16,186 genes (English and Butte, 2007). However, the mechanisms governing that regulation have remained unclear. To our knowledge, the current study is the first to demonstrate that TSPYL1 can influence the expression of cholesterol metabolizing CYPs, particularly CYP1B1, *via* Wnt/β-catenin signaling in HepaRG cells. We observed the binding of TCF7L2 to β-catenin and TSPYL1, and that β-catenin binding with TCF7L2 could be reduced by TSPYL1 overexpression. Those observations are compatible with a competition between TSPYL1 and TCF7L2 for binding to β-catenin. Although our CHIP assay showed that TCF7L2 and TSPYL1 bind to the same region of the *CYP1B1* promoter in HepaRG cells, we do not know whether TSPYL1 binds directly or indirectly to the *CYP1B1* promoter region by binding jointly with TCF7L2. Our group had already predicted the TSPYL1 DNA binding motif based on CHIP-PCR assay (Qin et al., 2018). That sequence is not the same as the TCF7L2 binding motif. However, there is a TCF7L2 binding motif (TCAAAG) in the *CYP1B1* promoter. As a result, it is possible that TSPYL1 may bind to the *CYP1B1* promoter as a result of its interaction with TCF7L2. Consistent with a previous publication (Malovannaya et al., 2011), our results suggest that, as transcriptional regulatory proteins, TSPYLs regulate gene transcription by interacting with transcription factors which bind with target gene promoter regions. A transcription factor (TF) is a sequence-specific DNA-binding factor. Specifically, it is a protein that controls the rate of transcription of genetic information from DNA to messenger RNA by binding to a specific DNA sequence. However, TSPYLs do not bind to a specific DNA sequence, as demonstrated by CHIP-PCR in our current and previous studies (Qin et al., 2018). Instead, transcription regulators influence transcriptional regulation by interacting with other proteins to “fine tune” transcription. As a result, TSPYLs are considered as transcription regulators, but not transcription factors.

In summary, the results of this series of studies indicates that TSPYL1 might be an important modulator of *CYP1B1* and, possibly, of *CYP7A1* expression in human hepatic cells. The identification of an “antagonistic” relationship between β-catenin and TSPYL1 adds a possible new layer of regulation to the complex network involving β-catenin, as well as the role of the TSPYL1 as an important regulator of CYP expression in the human liver. Finally, in summary, our findings suggest that decreased TSPYL1 expression might result in altered cholesterol metabolism through the regulation of cholesterol metabolizing CYPs in a process mediated by Wnt/β-catenin signaling.

## Data Availability

The original contributions presented in the study are included in the article/Supplementary Material, further inquiries can be directed to the corresponding author.

## Supplementary material

SUPPLEMENTARY FIGURE S1 The pie charts and Venn diagrams for each individual pull down by TSPYL1, 2 and 4 . Pie charts depicting the signaling pathways pulled down by TSPYL1 **(A)**, TSPYL2 **(B)**, or TSPYL4 **(C)**, respectively. **(D)** A Venn diagram of TSPYLs interacting signaling pathways for all three TSPYLs studied. The TSPYL interacting proteins and pathways are listed in a supplementary excel file.

SUPPLEMENTARY FIGURE S2 TSPYL1 binding to the *CYP7A1* promoter region in HepaRG cells was not detected. **(A)**Four pairs of primers covering the *CYP7A1* promoter region were used for CHIP assays. **(B)** CHIP assays were performed to test TSPYL1 binding to the *CYP7A1* promoter region using HepaRG cells. The binding of TSPYL1 to the *CYP7A1* promoter is shown as fold enrichment compared with IgG control. A set of control primers targeted to non- protein binding areas were used as a negative control. Compared with the negative control, TSPYL1 was not found to bind to the *CYP7A1* promoter region in HepaRG cells.

SUPPLEMENTARY FIGURE S3
**(A,B)** Effect of Wnt/*β*-catenin signaling on basal CYP expression. For *β*-catenin knockdown, HepG2 cells were treated with siRNA against *β*-catenin (C, blue bars). For *β*-catenin (CTNNB1) activation, HepG2 cells were treated for 24 h with 200 ng/ml Wnt-3a (D, red bars). The levels of the indicated CYPs were measured by PCR. By β-catenin knockdown in HepG2 cells, CYP1B1, CYP2A6 and CYP2B6 were transcriptionally downregulated more than 2-fold, whereas CYP2C9, CYP2C18, CYP3A7, CYP7A1, CYP7B1, and CYP27A1 were upregulated by β-catenin knockdown **(A)**. By contrast, after Wnt-3a stimulation, CYP1B1, CYP2A6, and CYP2B6 expression was significantly upregulated, while the expression of CYP2C9, CYP2C19, and CYP3A7 was downregulated more than 2-fold **(B)**. Vehicle controls (0.1% bovine serum albumin and nontargeting control siRNA, respectively) were used. Data represent means ± SD of three technical replicates per experiments. Student *t*-test were used for statistical analysis. Statistical significance is indicated by asterisks. *P < 0.05, **P < 0.01, ***P < 0.001, ****P < 0.0001.

SUPPLEMENTARY TABLE S1 Summary of mass spectrometric analysis of wnt signaling proteins associated with TSPYLs.

SUPPLEMENTARY TABLE S2 ChIP-qPCR primer sets for promoter regions of *CYP1B1* and *7A1*.
