# Supplementary material for: Testis- specific Y-encoded- like protein 1 and cholesterol metabolism: Regulation of CYP1B1 expression through Wnt signaling
Source: Front Pharmacol. 2022 Nov 28;13:1047318. doi: 10.3389/fphar.2022.1047318 (PMC9742362; doi:10.3389/fphar.2022.1047318)
Supplement: Supplementary file 1 [file DataSheet2.pdf]

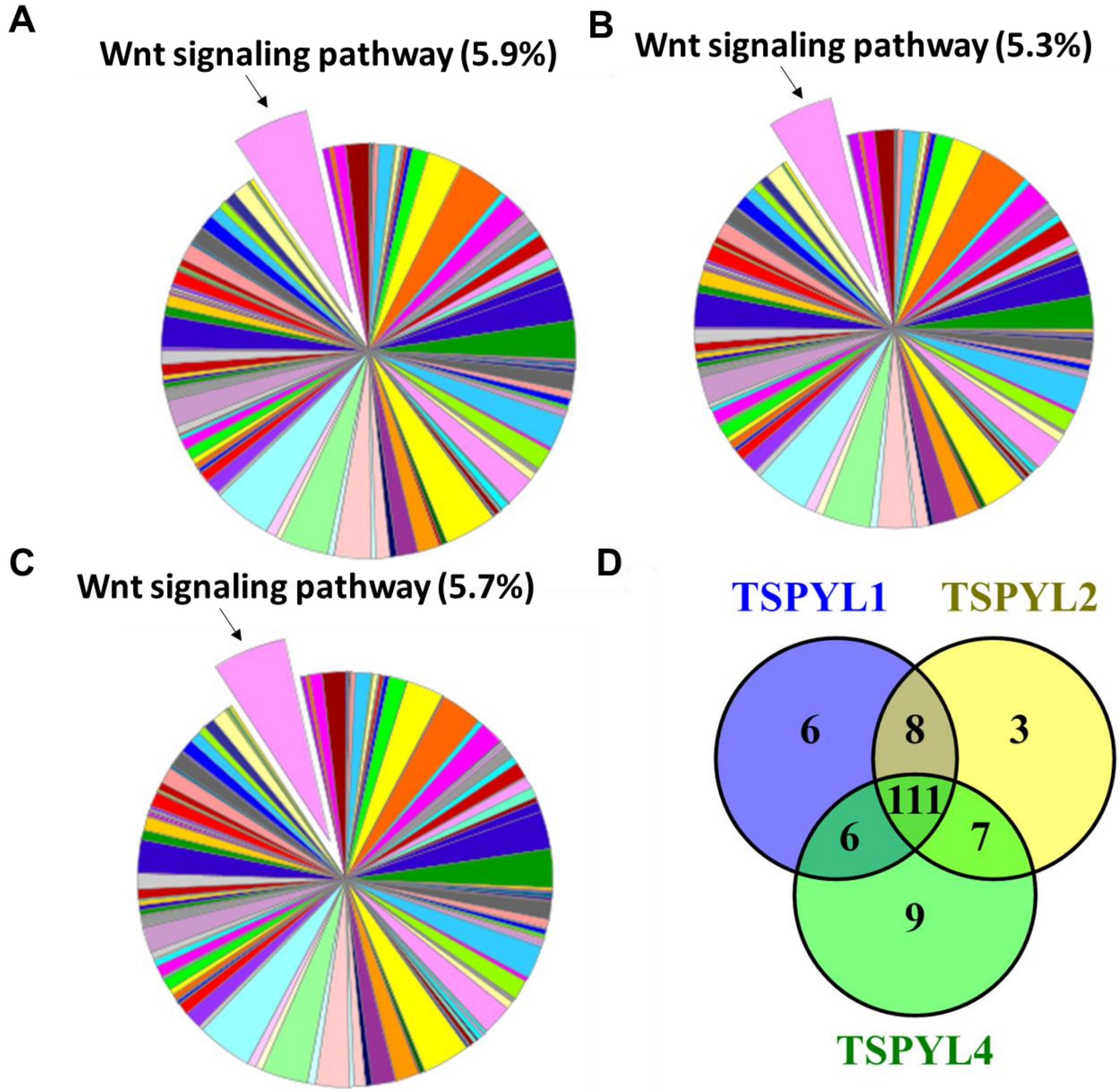

Supplementary Figure 1. The pie charts and Venn diagrams for each individual pull down by TSPYL1, 2 and 4 (A, B, C). Pie charts depicting the signaling pathways pulled down by TSPYL1 (**A**), TSPYL2 (**B**), or TSPYL4 (**C**), respectively. (**D**) A Venn diagram of TSPYLs interacting signaling pathways for all three TSPYLs studied. The TSPYL interacting proteins and pathways are listed in a supplementary excel file.

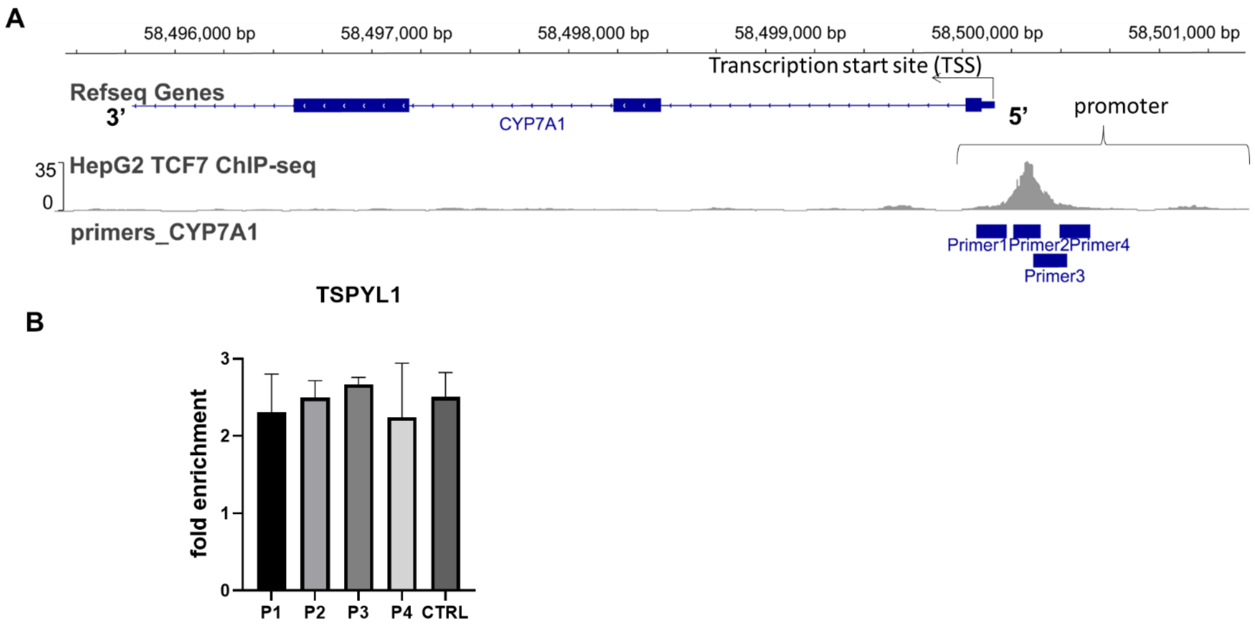

Supplementary Figure 2. TSPYL1 binding to the *CYP7A1* promoter region in HepaRG cells was not detected. (A) Four pairs of primers covering the *CYP7A1* promoter region were used for CHIP assays. (**B**) CHIP assays were performed to test TSPYL1 binding to the *CYP7A1* promoter region using HepaRG cells. The binding of TSPYL1 to the *CYP7A1* promoter is shown as fold enrichment compared with IgG control. A set of control primers targeted to non- protein binding areas were used as a negative control. Compared with the negative control, TSPYL1 was not found to bind to the *CYP7A1* promoter region in HepaRG cells.

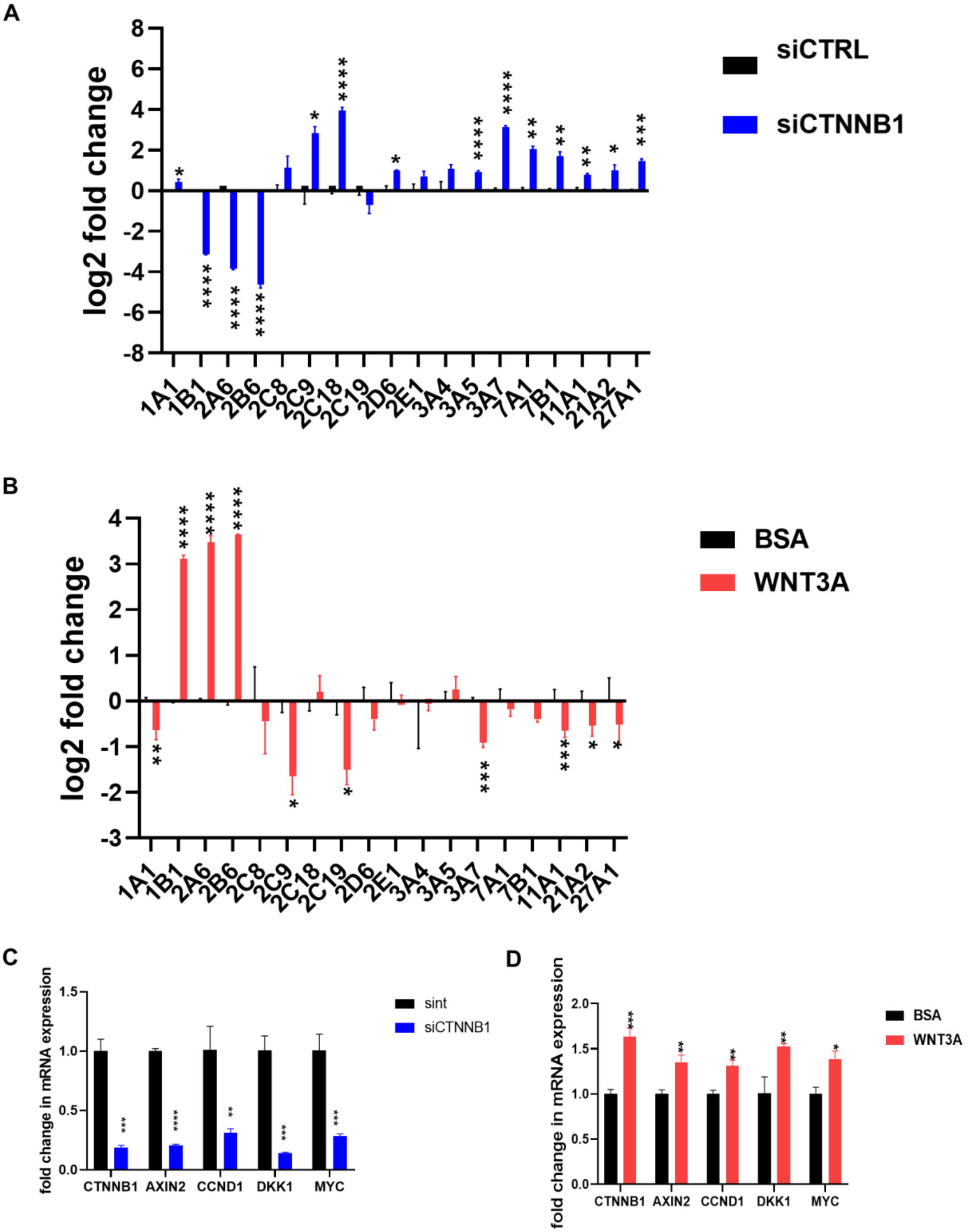

Supplementary Figure 3. (**A, B**) **Effect of Wnt/*β*-catenin signaling on basal CYP expression.** For *β*-catenin knockdown, HepG2 cells were treated with siRNA against *β*-catenin (**C**, blue bars). For *β*-catenin

(CTNNB1) activation, HepG2 cells were treated for 24 hours with 200 ng/ml Wnt-3a (**D**, red bars). The levels of the indicated CYPs were measured by PCR. By β-catenin knockdown in HepG2 cells, CYP1B1, CYP2A6 and CYP2B6 were transcriptionally downregulated more than 2-fold, whereas CYP2C9, CYP2C18, CYP3A7, CYP7A1, CYP7B1, and CYP27A1 were upregulated by β-catenin knockdown (**A**). By contrast, after Wnt-3a stimulation, CYP1B1, CYP2A6 and CYP2B6 expression was significantly upregulated, while the expression of CYP2C9, CYP2C19 and CYP3A7 was downregulated more than 2-fold (**B**). Vehicle controls (0.1% bovine serum albumin and nontargeting control siRNA, respectively) were used. Data represent means ± SD of three technical replicates per experiments. Student *t*-test were used for statistical analysis. Statistical significance is indicated by asterisks. *P < 0.05, **P < 0.01, ***P < 0.001, ****P<0.0001.

Supplemental Tables S1

Table. S1 Summary of mass spectrometric analysis of wnt signaling proteins associated with TSPYLs.

**Wnt signaling proteins**								
ACTBL2	CDH2	CTNNA2	FZD3	MMP7	PCDH7	PLCB1	SIAH2	TP53
ACTC1	CDH23	CTNNA3	FZD6	MYC	PCDH8	PLCB2	SMAD4	WNT1
ACVR1C	CDH3	CTNNAL1	FZD7	MYH1	PCDH9	PLCB3	SMARCA1	WNT10A
ANKRD6	CDH3	CTNNB1	FZD8	MYH13	PCDHA10	PLCB4	SMARCA2	WNT10B
APC	CDH8	DACT1	GNA15	MYH15	PCDHA12	PPP2CB	SMARCA4	WNT11
APC2	CDHR1	DCHS1	GNB3	MYH3	PCDHA2	PPP2R5C	SMARCA5	WNT16
ARID1A	CDHR3	DCHS2	GNG12	MYH6	PCDHA3	PPP2R5D	SMARCC1	WNT2
ARID1B	CELSR1	DVL1P1	GNG2	MYH7	PCDHA7	PPP3CB	SMARCC2	WNT2B
ARRB1	CELSR2	DVL2	HDAC2	MYH7B	PCDHAC1	PRKCA	SMARCD1	WNT3
ARRB2	CELSR3	EN2	HDAC8	NFATC1	PCDHAC2	PRKCB	SMARCD2	WNT4
AXIN1	CHD1L	EP300	HLTF	NFATC2	PCDHB13	PRKCD	SPINK6	WNT5B
AXIN2	CREBBP	EP400	INO80	NFATC3	PCDHB14	PRKCG	SRCAP	WNT6
BCL9	CSNK1A1	FAT1	ITPR1	NFATC4	PCDHGA1	PRKCH	TBL1X	WNT7A
BMPR1A	CSNK1D	FAT2	ITPR2	NKD1	PCDHGA12	PRKCI	TCF3	WNT7B
BTRC	CSNK1E	FAT3	ITPR3	NLK	PCDHGA3	PRKCQ	TCF7	WNT8A
CDH11	CSNK1G1	FAT4	KREMEN2	PCDH1	PCDHGA5	PRKCZ	TCF7L2	WNT8B
CDH13	CSNK1G3	FRZB	LRP5	PCDH12	PCDHGA8	PYGO2	TLE2	WNT9B
CDH15	CSNK2B	FSTL1	LRP5L	PCDH15	PCDHGB1	SDK1	TLE3	
CDH16	CTBP1	FZD10	LRP6	PCDH19	PCDHGB6	SFRP4	TLE4	
CDH18	CTNNA1	FZD3	MAP3K7	PCDH20	PCDHGC3	SIAH1	TLE6	

Supplemental Tables S2

Table. S2 ChIP-qPCR primer sets for promoter regions of *CYP1B1* and *7A1*

Gene	Sequences
***CYP1B1*-P1**	**Forward:** ACACCAGGCCGCTTTGACCC<br>**Reverse:** GGCCTCGATTGGAGGTGGCTGT
***CYP1B1*-P2**	**Forward:** CGCTTCATCACAGCCACCTCCAA<br>**Reverse:** TCCGGGAAGCAAGCTCAAGTCG
***CYP1B1*-P3**	**Forward:** TCCGCGACTTGAGCTTGCTTCC<br>**Reverse:** TGAGGTGGCAATTTGTTTGCGAGA
***CYP1B1*-P4**	**Forward:** TTGCCACCTCAGTGGAGGCTCTTT<br>**Reverse:** GGAGTGGCCTCTACGCGGGAAAT
***CYP1B1*-P5**	**Forward:** CCTGAGATTTCCCGCGTAGAGGC<br>**Reverse:** CCTGCGCCAACGGCTTCCAT
***CYP1B1*-P6**	**Forward:** CACCGGCGGCTCTGTGGTCT<br>**Reverse:** CCCGCCTCGTGAAGTCCTTGTTC
***CYP1B1*-P7**	**Forward:** CGAGGGAAGAGGTGGGATGTATCTG<br>**Reverse:** TCATCTGTAAACAGGTCAATAATAGCACGA
***CYP7A1*-P1**	**Forward:** CTCTGGCAAAGCACCTAAAT<br>**Reverse:** CTGTTTAAGATGGGCATAGC
***CYP7A1*-P2**	**Forward:** CAGGTCCGAATGTTAAGTCA<br>**Reverse:** AGTAACTGGCCTTGAACTAA
***CYP7A1*-P3**	**Forward:** TAGTTCAAGGCCAGTTACTACCA<br>**Reverse:** AAGGGAAGGATGCCACTGAA
***CYP7A1*-P4**	**Forward:** TTCAGTGGCATCCTTCCCTT<br>**Reverse:** CCAAGAATAAGCCATAGACAA
